# A modular tool to query and inducibly disrupt biomolecular condensates

**DOI:** 10.1038/s41467-021-22096-1

**Published:** 2021-03-22

**Authors:** Carmen N. Hernández-Candia, Sarah Pearce, Chandra L. Tucker

**Affiliations:** Department of Pharmacology, University of Colorado School of Medicine, Aurora, CO USA

**Keywords:** Biochemistry, Molecular engineering, Optogenetics, Intrinsically disordered proteins, Synthetic biology

## Abstract

Dynamic membraneless compartments formed by protein condensates have multifunctional roles in cellular biology. Tools that inducibly trigger condensate formation have been useful for exploring their cellular function, however, there are few tools that provide inducible control over condensate disruption. To address this need we developed DisCo (Disassembly of Condensates), which relies on the use of chemical dimerizers to inducibly recruit a ligand to the condensate-forming protein, triggering condensate dissociation. We demonstrate use of DisCo to disrupt condensates of FUS, associated with amyotrophic lateral sclerosis, and to prevent formation of polyglutamine-containing huntingtin condensates, associated with Huntington’s disease. In addition, we combined DisCo with a tool to induce condensates with light, CRY2olig, achieving bidirectional control of condensate formation and disassembly using orthogonal inputs of light and rapamycin. Our results demonstrate a method to manipulate condensate states that will have broad utility, enabling better understanding of the biological role of condensates in health and disease.

## Abstract (web summary)

Here, the authors present DisCo (Disassembly of Condensates), a method that allows the fast, inducible, and specific disruption of tagged condensates in mammalian cells. DisCo uses chemical dimerizers to induce the recruitment of a ligand into condensates leading to condensate disassembly.

## Introduction

Membraneless organelles are biomolecular condensates formed by proteins that undergo a phase transition, unmixing the protein solution into two phases with different concentrations, a dense phase and a bulk dilute phase. Condensate formation involves the association of multivalent proteins or proteins carrying low complexity regions (LCRs), and can also involve interaction with RNA molecules^1^. In the cell, membraneless organelles include structures such as stress granules, p-bodies, photobodies, and the nucleolus, where multiple proteins associate in a highly concentrated compartment without the need of a physical barrier^1,2^. The absence of a physical barrier makes cellular condensates highly dynamic: proteins can rapidly diffuse in and out, and likewise the condensate itself can rapidly form and dissociate. Loss of the dynamic character of cellular condensates, wherein proteins that form liquid-like condensates can further evolve into more rigid structures, is associated with an impaired response to stress and the development of neurodegenerative diseases^2–4^. A better understanding of the mechanisms that control the formation and disassembly of biomolecular condensates is important not only for elucidating the biological roles of membraneless organelles, but for the development of new treatments for pathologies associated with aberrant phase transitions.

Tools that allow the assembly and disassembly of condensates at user-specified times are valuable for researchers seeking to understand their dynamic properties and biological functions. A number of molecular tools have been developed that, either alone or when fused to condensate-forming proteins, allow inducible control of the assembly of biomolecular condensates actuated by light^5–10^ or chemicals^9,11^. Light-induced oligomerizers have been used to synthetically induce biomolecular condensate states relevant to neurodegenerative disease, by fusing to full-length or domains of FUS, TDP-43, and G3BP1^8,12–14^. These methods have been highly useful for elucidating the natural roles of condensate structures within cells and to better understand the contribution of factors such as multivalency and LCRs^8–10,15^. Tools that artificially induce clustered or condensate states have also been used to control protein function in unique ways, for example, to regulate signal transduction^16–18^, control endocytosis^5^, regulate cellular metabolism^19^, reconstitute RNA granules^9^ or nuclear bodies^11^, and bring together targeted genomic loci^15^.

While much work has focused on methods to induce condensate formation, evidence suggests that condensate disassembly is equally dynamic, and can be tightly regulated by protein-RNA^12^ or protein–protein interactions. In several examples of naturally occurring condensate disruption, disassembly involves the new interaction of a “client protein” (protein not involved in forming the condensate) with the condensate-forming protein (hereafter referred to as “scaffold”). The recruited client ligand binds at a specific site on the scaffold, leading to condensate disassembly or blocking of condensate formation. For example, the formation of condensates of FUS is inhibited by binding of karyopherin-β2, a high-affinity binding partner that interacts with the FUS proline-tyrosine-rich nuclear localization sequence^20–22^. Likewise, profilin reduces aggregation of exon 1 of huntingtin protein (HTTex1) by directly binding its proline-rich region^23^. Using a synthetic approach, intrabodies directed to the N-terminus of huntingtin have been shown to block the formation of fibrillar aggregates^24^. In addition to protein binding, post-translational modifications can also affect condensate states. The addition of ubiquitin modifications to ubiquilin UBQLN2 condensates resulted in condensate disassembly, while the phosphorylation of FUS LCRs resulted in reduced FUS condensate formation and decreased toxicity^25,26^. In all these examples, condensate states of proteins were disrupted by binding or modification directly within the domain involved in condensate assembly. It remains an open question whether direct interaction within the condensate assembly domain is essential for disassembly. In a recent computational modeling study, the binding of a monovalent ligand to a ‘spacer’ domain distinct from the domain mediating assembly was found to promote condensate disassembly^27^. However, there have been few studies experimentally testing whether and how a ligand that binds a condensate-forming scaffold triggers growth or dissolution.

In addition to disassembly triggered by protein binding or modification, several other methods have been developed to trigger condensate disruption. Liquid-like condensates can be dissolved by the chemical 1,6-hexanediol, presumably acting through the disruption of hydrophobic interactions^28^. While this chemical can be useful for determining if a protein exists in a condensate state, its extended use leads to loss of membrane integrity and cell death^28^. A less toxic small molecule, lipoic acid, was recently reported to reduce the propensity of stress granule proteins to aggregate^29^; however, it only targets stress granules and cannot be used with other condensates. A strategy to disrupt condensates using light, PixELL (Pix Evaporates from Liquid-like droplets in Light)^30^, involves reducing the multivalency of the condensate-forming protein. While PixELL provides a useful tool to understand the spatial regulation of biomolecular condensates, the condensates are created by the combination of an LCR and a multivalent scaffold, and thus the tool is not well-suited to probe the biological consequences of disrupting condensates formed by the native, full-length protein.

**Fig. 1:**
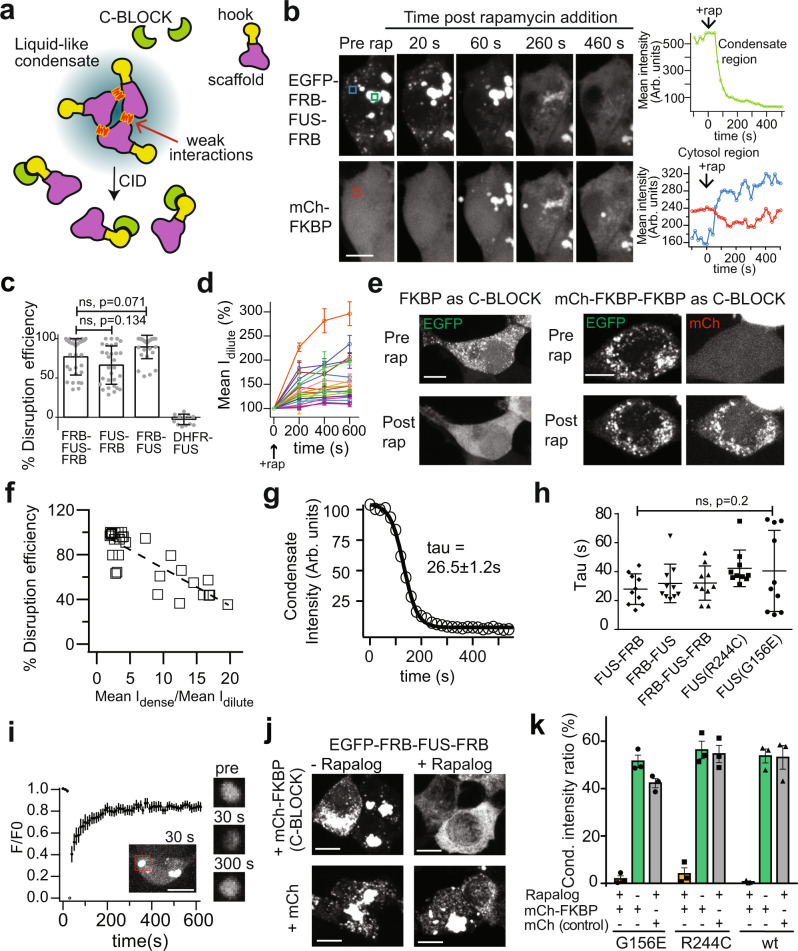
DisCo can be used to disrupt and prevent formation of FUS condensates. **a** General overview of DisCo approach with liquid-like condensates. A ligand (C-BLOCK) is recruited by chemical-induced dimerization (CID) to bind to a “hook” domain on a scaffold that forms the condensate. Binding results in destabilization of the weak interactions that hold together the condensate and condensate dissolution. **b** Representative images of HEK293T cells transfected with EGFP-FRB-FUS-FRB, along with the C-BLOCK mCh-FKBP. Addition of 333 nM rapamycin induced fast disruption of FUS condensates. Graphs show the mean fluorescent intensities at the indicated regions in timelapse images for FUS in condensate (green, top right), FUS in dilute phase (blue, bottom right) and mCh-FKBP (red, bottom right). Scale bar, 10 µm. Arb. units, arbitrary units. Similar results were obtained in 5 independent repeats. **c** Recruitment of C-BLOCK to different orientations within FUS condensates does not affect the % disruption efficiency, defined as the % of initial condensate that is disrupted upon rapamycin addition. HEK293T cells expressing indicated constructs were monitored for ability of C-BLOCK recruitment to disrupt condensate states. Data shows average and error (s.d., *n* = 30 for EGFP-FRB-FUS-FRB, EGFP-FUS-FRB, and EGFP-FRB-FUS, and *n* = 12 for EGFP-DHFR-FUS). ns, not significant, Kolmogorov–Smirnov (two-tailed) test, *p* = 0.071(FRB-FUS-FRB vs. FRB-FUS) and *p* = 0.134 (FRB-FUS-FRB vs. FUS-FRB). **d** Quantification of change in FUS dilute phase signal after rapamycin addition. HEK293T cells expressing EGFP-FRB-FUS-FRB and mCh-FKBP were treated with 333 nM rapamycin as in (**b**). After addition, an increase in EGFP signal outside of the condensate is observed. Graphs show the normalized mean fluorescent intensity of EGFP in the dilute phase (cytosol) at three time points after rapamycin addition. Graph shows average and error (s.e.m) of three measurements for each cell, *n* = 20 cells from three separate experiments. **e** HEK293T cells expressing EGFP-FRB-FUS-FRB and indicated C-BLOCKs were treated with rapamycin as in Fig. 1b. Condensates were disrupted upon recruitment of FKBP, but not mCh-FKBP-FKBP. Representative images from a single experiment are shown; experiments were repeated twice with similar results. Scale bars, 10 µm. **f** For cells expressing EGFP-FRB-FUS-FRB and mCh-FKBP the % disruption efficiency was plotted with respect to a value representing the ratio of GFP signal (mean intensity) within condensates to signal in the dilute phase (Mean I_dense_/Mean I_dilute_). A correlation was observed between the degree of condensate disruption, and the Mean I_dense_/Mean I_dilute_ value. **g** Disruption kinetics of a representative cell expressing EGFP-FRB-FUS-FRB and mCh-FKBP treated with rapamycin (333 nM). The amount of FUS protein within condensates as a function of time was quantified, then fit to a sigmoid function to obtain the characteristic time (tau). Arb. units, arbitrary units. **h** Characteristic time (tau) of DisCo-mediated FUS disruption for indicated constructs. Mutant FUS constructs contained the configuration EGFP-FRB-FUS-FRB. Data shows average and error (s.d., *n* = 10 cells examined over three independent experiments). ns, not significant (*p* = 0.02), non-parametric one-way ANOVA. **i** Fluorescence recovery after photobleaching (FRAP) analysis of EGFP-FRB-FUS-FRB condensates. Representative images of a photobleached condensate are shown at right. Graph shows average and error (s.e.m., *n* = 11 cells examined over three independent experiments). Scale bars, 10 µm. **j** Preemptive recruitment of C-BLOCK to FUS blocks FUS condensate formation. Representative images of HEK293T cells expressing EGFP-FRB-FUS-FRB and indicated C-BLOCK or mCherry control are shown. Cells were treated with 500 nM AP21967 (+Rapalog) for 18 h, added 4 h after transfection, and imaged at 22 h post-transfection. The experiment was repeated 3 times with similar results. Scale bars, 10 µm. **k** Quantification of FUS signal in condensates 22 h post-transfection, with or without prior C-BLOCK recruitment. HEK293T cells expressing EGFP-FRB-FUS-FRB and mCh-FKBP (or mCh as control) were incubated with 500 nM AP21967 (+rapalog) prior to protein expression (as in 1j). The condensate intensity ratio was determined by measuring the integrated intensity within all condensates in a maximum projection image normalized to the total integrated intensity for all the cells in the maximum projection image. Data shows average and error (s.e.m., *n* = 3 regions with cells examined from three independent experiments).

Here we describe a method named DisCo (Disassembly of Condensates), that uses chemicals to rapidly and inducibly trigger disassembly of user-specified condensates. The method uses chemical-induced dimerization (CID) to recruit a ligand (termed ‘C-BLOCK’) to the scaffold protein, resulting in the disruption of the condensates, and requires only a small, 11 kDa ‘hook’ attachment to the scaffold protein (Fig. 1a). While the method works well in dissociating preformed liquid-like condensates, it also can be applied before condensates are formed, preventing the assembly of both liquid-like and solid-like condensates. We validated the DisCo approach with disease-relevant condensates formed by FUS, associated with familial amyotrophic lateral sclerosis (ALS) and frontotemporal lobal degeneration (FTLD)^31^, and an expanded polyglutamine (72Q)-containing huntingtin Exon 1 associated with Huntington’s disease pathology^32^. DisCo was able to disrupt and block the formation of FUS condensates and prevent the formation of more solid-like huntingtin condensates. DisCo can also be used to disrupt optogenetic condensates formed by CRY2olig^5^, enabling rapid, orthogonal, and bidirectional control over their assembly and disassembly. Together, our studies show DisCo provides an additional layer of control over natural and synthetic condensates, which should be useful for probing a variety of condensate-driven cellular processes.

## Results

### FUS condensates can be disrupted or inhibited using DisCo

The liquid-like unmixed state of biomolecular condensates is maintained by a network of weak interacting forces that occur between LCRs or multivalent proteins. If stronger interacting forces are involved, the biomolecular condensates become more rigid and less dynamic, behaving as gels or solids^2,33^. We rationalized that recruitment of a ligand (C-BLOCK) to the scaffold protein that forms the condensates could induce its dissolution, as has been previously observed with natural binding proteins^20–22^. To trigger recruitment of a C-BLOCK protein to condensates, we used a rapamycin-based CID system, which mediates high-affinity dimerization of FRB and FKBP (Fig. 1a).

To test the DisCo approach, we first focused on condensates formed by the DNA/RNA binding protein FUS, involved in RNA processing and DNA repair^34,35^. Disease-associated variants of FUS are implicated in neuropathologies such as ALS and FTLD^31^, where they result in abnormal inclusions in the brain. Prior studies have shown that FUS forms condensates that undergo a protein phase transition from a more dynamic liquid-like state to a more solid-like state, and that this transition occurs faster in ALS disease-associated variants^4^. To examine the ability of DisCo to disrupt FUS condensates, we attached EGFP and FRB to full-length FUS (EGFP-FRB-FUS-FRB), using a T2098L variant of FRB that can bind both rapamycin and the rapalog AP21967^36^. For a C-BLOCK, we fused a single domain of FKBP12 to mCherry (mCh-FKBP), enabling tracking. Upon addition of the chemical dimerizer rapamycin, we expected that the C-BLOCK would be recruited to FUS. As rapamycin is known to have adverse effects on cells through inhibition of mTor^37^, our studies used rapamycin only for short-term experiments, with the orthogonal rapalog AP21967 substituted for longer-term studies.

We overexpressed wild type FUS constructs at high levels in HEK293T cells to drive the formation of condensates, thus enabling better visualization of condensate disruption. When expressed in HEK293T cells, a version of FUS containing two FRB “hooks” (EGFP-FRB-FUS-FRB) formed condensates (Fig. 1b, “Pre rap”). Addition of 333 nM rapamycin to induce recruitment of mCh-FKBP to FUS resulted in rapid dissolution of the FUS condensate (Fig. 1b, c and Supplementary Movie 1), with a concomitant increase of EGFP-FRB-FUS-FRB signal in the dilute phase (diffuse cytosolic signal) (Fig. 1d). Dissolution required the presence of the FRB hook, but was not dependent on the hook orientation, as FUS condensates with FRB attached at different configurations (EGFP-FUS-FRB, EGFP-FRB-FUS) were equivalently disrupted (Fig. 1c). To exclude that the loss of EGFP signal was not just due to FRET (förster resonance energy transfer) caused by induced proximity of EGFP and mCherry, we also tested a non-fluorescent K70N mCherry-FKBP as C-BLOCK, which gave similar results (Supplementary Fig. 1 and Supplementary Movie 2). A minimal C-BLOCK, consisting of a single FKBP domain, was also effective at disrupting condensates, while a multivalent mCh-FKBP-FKBP was not disruptive (Fig. 1e). These preliminary results suggested that the valency, rather than size, of the C-BLOCK could be an important criterion for disruption.

While many of the condensates were completely disrupted, others showed intermediate levels of disruption (between 35-90% disruption efficiency, Fig. 1c). We examined whether the condensates that were not completely disrupted had higher concentrations of FUS scaffold in the dense phase compared with the dilute phase. As shown in Fig. 1f, the disruption efficiency showed a linear correlation, with condensates more effectively disrupted in cells with lower ratios (i.e., less separation between dense and dilute phases). Disruption of condensates showed a sigmoid behavior, with a time constant (τ, tau) of less than a minute (Fig. 1g), and was equally effective with both wild-type FUS and disease-associated variants (G156E and R244C) known to accelerate transition into fiber-like states^4^ (Supplementary Fig. 2). Disruption of condensates formed by all FRB-FUS tested combinations occurred within a similar time frame (τ, 25-45 s) (Fig. 1h). To test the dynamic nature of condensates, we performed fluorescence recovery after photobleaching (FRAP) experiments (Fig. 1i), observing that FUS condensates showed dynamic exchange with the cytosol. We also validated our results in a lower-expression COS-7 system, where FUS condensates formed in response to arsenite-induced stress were also disrupted by rapamycin addition (Supplementary Fig. 3).

To test if prior recruitment of a C-BLOCK protein could prevent FUS condensate formation, we transfected cells with EGFP-FRB-FUS-FRB (wt, G156E, or R244C) and mCh-FKBP and added the rapalog AP21967 to cells immediately after transfection, prior to protein expression. The preemptive recruitment of C-BLOCK to FUS prevented condensate formation with both wt and mutant variants (Fig. 1j, k and Supplementary Fig. 2b). Control cells coexpressing EGFP-FRB-FUS-FRB and mCh (without FKBP) and incubated with AP21967 were not blocked from forming condensates (Fig. 1k).

**Fig. 2:**
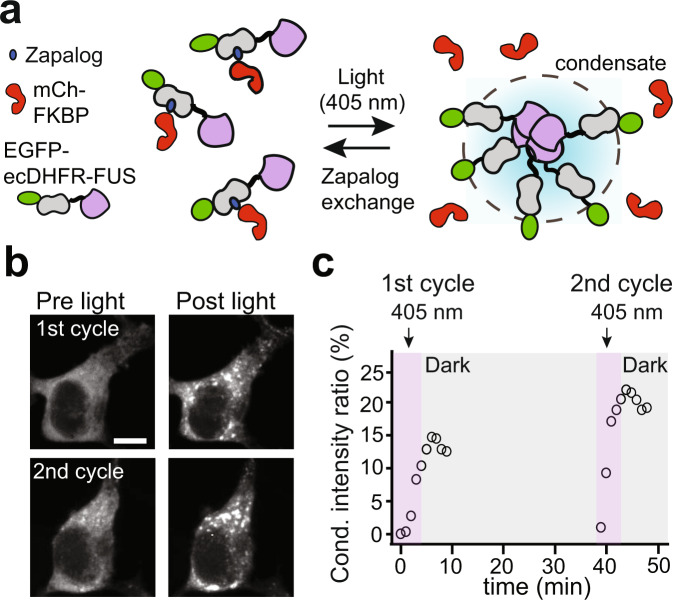
Use of DisCo with a photocleavable dimerizer allows reversible control of FUS condensate formation and disruption. **a** Schematic showing use of the photocleavable heterodimerizer zapalog to reversibly control FUS condensate disruption. **b** Representative cell expressing EGFP-ecDHFR-FUS and mCh-FKBP incubated with 500 nM zapalog. Prior to illumination, FUS condensates are prevented from formation by C-BLOCK binding. Approximately 2–3 min after illumination with 405 nm light (50 ms pulse every 30 s for 5 min), FUS condensates can clearly be observed. After light treatment, incubation with zapalog in the dark for 34 min allows binding of uncleaved zapalog from the media and restores condensate blocking effect. A second round of 405 nm illumination induced further FUS condensate formation. The experiment was repeated an additional time with similar results. Scale bar, 10 µm. **c** Quantification of EGFP-ecDHFR-FUS condensate formation for experiment in (**b**), with data shown from a single experiment.

We next examined if DisCo could be configured to allow multiple rounds of condensate dissolution. To test this, we used a different chemical dimerizer, zapalog, consisting of trimethoprim fused to FK506 by means of a photocleavable linker^38^. Uncleaved zapalog mediates interaction between FKBP12 and bacterial DHFR, while exposure to a short 405 nm light pulse cleaves zapalog and results in rapid dissociation of the dimerized proteins. This effect is further reversible as the cleaved probe exchanges with uncleaved zapalog in the media within minutes, restoring the FKBP/DHFR interaction^38^. We envisioned zapalog could allow constitutive recruitment of C-BLOCK to FUS, preventing condensate formation, but that addition of 405 nm light would dissociate C-BLOCK, allowing condensates to form for the duration of light application (Fig. 2a). Such an approach would provide reversibility and spatial control over condensate disruption. Indeed, HEK293T cells coexpressing EGFP-DHFR-FUS and mCh-FKBP exposed to 500 nM zapalog did not initially form FUS condensates. However, the addition of 405 nm light (50 ms pulse every 1 min for 5 min) triggered the formation of FUS condensates, first observed within 2 min (Fig. 2b, c). After condensates had formed by light application, we incubated cells in the dark to allow exchange of the photocleaved zapalog for uncleaved compound, allowing the protein to return to the soluble state. A second round of 405 nm light treatment (after 34 min in dark) induced a second round of FUS condensation (Fig. 2b, c), indicating the capacity for multiple rounds of condensate formation and dissolution and generality of the system for use with other induced dimerization systems.

### Inhibition of Huntingtin Exon 1 condensates using DisCo

**Fig. 3:**
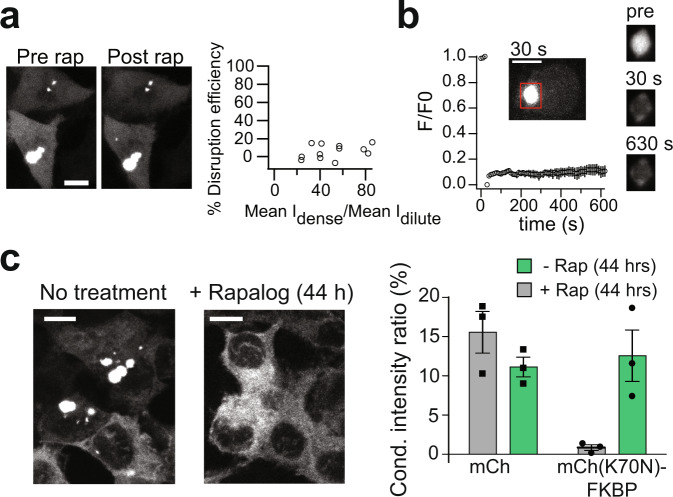
HTT exon 1 condensates can be prevented from formation using DisCo but existing condensates are not disrupted. **a** Representative images (left) and quantification of % disruption efficiency as a function of the initial EGFP mean signal in the dense phase normalized to the EGFP mean signal at the dilute phase ((Mean I_dense_)/(Mean I_dilute_)) (right) of HEK293T cells coexpressing HTTQ72-FRB-EGFP and mCh(K70N)-FKBP as C-BLOCK, exposed to 333 nM rapamycin. The experiment was repeated 3 times with similar results. Scale bars, 10 µm. **b** FRAP experiments showing Q72HTT-FRB-EGFP condensates do not recover from photobleaching within 600 s. Data shows average and error (s.e.m., *n* = 8). Representative images of a photobleached condensate are shown at right. Scale bars, 10 µm. **c** Preemptive recruitment of C-BLOCK to HTT prevents condensate formation. HEK293T cells coexpressing HTTQ72-FRB-EGFP and mCh(K70N)-FKBP were treated for 44 h with 500 nM AP21967 rapalog. Treated cells show a reduction in HTTex1 condensates compared with untreated cells or cells not expressing C-BLOCK. Representative images are shown at left, with quantification (as in Fig. 1k) at right. Data represents average and error (s.e.m, *n* = 3 regions with cells examined from 3 independent experiments). Scale bars, 10 µm.

To validate DisCo with a different type of condensate, we tested the approach with exon 1 of huntingtin protein (HTTex1), containing a pathological 72Q expanded polyglutamine repeat. HTTex1 carries at its N-terminus a LCR formed by a polyglutamine tract of variable length and a proline-rich motif, which was recently linked to the capability of HTTex1 to form condensates that evolved from a liquid-like state into a fibrillar aggregated state^39^. We fused HTT(Q72)ex1 to FRB and EGFP (e.g., HTTQ72-FRB-EGFP) and expressed in HEK293T cells, along with K70N mCherry^40^ (mCh(K70N)-FKBP) as C-BLOCK. For cells forming condensates, the majority of EGFP signal was initially found in condensates (dense phase), with much lower signal in the bulk dilute phase of the cytosol. We observed minimal to no rapamycin-induced decrease of HTTQ72-FRB-EGFP signal in condensates, indicating these condensates could not be dissolved (Fig. 3a). FRAP experiments indicated that HTTQ72-FRB-EGFP condensates are unable to recover from photobleaching and have limited mobility (Fig. 3b), indicating a solid-like structure consistent with prior studies^39^.

Although DisCo was ineffective in disrupting existing HTTex1 condensates, we could use the method to prevent the formation of HTTex1 condensates in the first place. Cells transfected with HTTQ72-FRB-EGFP and mCh(K70N)-FKBP and treated preemptively with rapalog AP21967 just after transfection showed minimal condensate formation, as compared to cells lacking rapalog or the C-BLOCK (Fig. 3c). Taken together, our results indicate that DisCo provides a suitable strategy for controlled disruption of liquid-like condensates (for example formed by FUS), and can be used to prevent the formation of liquid-like condensates and their maturation into solid condensates, such as those formed by HTTex1.

### CRY2olig condensates can be formed with light and disrupted using DisCo

Our initial studies showed that DisCo was effective on condensates formed through LCRs, but did not examine efficacy with condensates formed through multivalent or oligomeric interactions. To examine this, we tested the effect of DisCo on condensates of CRY2, an *Arabidopsis* blue light photoreceptor that undergoes oligomerization upon exposure to blue light. For our studies, we used CRY2olig, a mutant truncated form of CRY2 that undergoes pronounced light-triggered oligomerization and forms puncta within cells^5^. In previous studies, light-triggered oligomerization of CRY2 and CRY2olig was used to modulate a variety of cellular activities, including kinase signaling states, small GTPase activity, actin polymerization, and endocytosis^5–7,16,17^. Activities induced by formation of CRY2 and CRY2olig condensates can be reversed in dark, as photostimulated CRY2 reverts back to its dark, non-oligomerized state^5–7^; however, full reversal can take up to tens of minutes. We envisioned that the use of DisCo could speed up the reversion process, allowing fast, induced reversal of activities modulated by CRY2 and CRY2olig clustering.

**Fig. 4:**
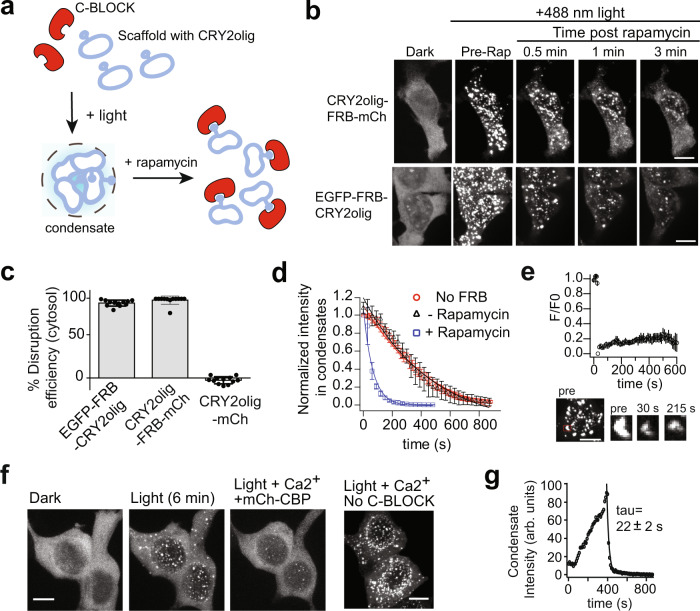
Light-activated CRY2olig condensates can be disrupted using DisCo. **a** Schematic of DisCo approach with CRY2olig. Light triggers condensate formation, while rapamycin-mediated recruitment of C-BLOCK to a FRB ‘hook’ on CRY2olig scaffold disrupts condensates. **b**, **c** CRY2olig condensates are disrupted equally regardless of recruitment orientation. DisCo was performed on HEK293T cells expressing CRY2olig-FRB-mCh and mCh(K70N)-FKBP, or EGFP-FRB-CRY2olig and mCh-FKBP. Cells were illuminated with light (488 nm, 100 ms every 30 s) at 18–22 h post-transfection, then 333 nM rapamycin was added 5 min after light onset. Representative images shown in (**b**), with quantification of cytosolic disruption in (**c**). Data were quantified as in Fig. 1c and represent average and error (s.d., *n* = 10 cells examined from three independent experiments). Scale bars, 10 µm. **d** Kinetics of condensate disassembly. HEK293T cells expressing CRY2olig-FRB-mCh and mCh(K70N)-FKBP were illuminated 5 min (488 nm, 100 ms pulse every 20–30 s), 18–22 h after transfection. Black triangles, dark, no rapamycin. Blue squares, light (488 nm, 100 ms pulse every 30 s), 333 nM rapamycin. Red circles, control cells expressing CRY2olig-mCh (no FRB) and mCh(K70N)-FKB, with 333 nM rapamycin in dark. All experiments were performed at 33.5 °C. Data shows average and error (s.d., n = 5 cells examined from three independent experiments). **e** FRAP experiments with EGFP-FRB-CRY2olig condensates formed after 3 min light treatment. Data shows average and error (s.e.m, *n* = 6 cells examined from three independent experiments). Representative images of a photobleached condensate are shown at bottom. Scale bars, 10 µm. **f** Ca^2+^-dependent DisCo. HEK293T cells expressing EGFP-CaM-CRY2oligC9 and mCh-CBP formed condensates with light (488, 100 ms pulse every 30 s for 6 min). Addition of 2.5 mM CaCl_2_ and 3 µM ionomycin resulted in condensate disruption. Image at far right shows control experiments subject to the same light and Ca^2+^ treatments, but without C-BLOCK. The experiment was repeated a second time with similar results. Scale bars, 10 µm. **g** Quantification of kinetics and extent of Ca^2+^-dependent disruption of CRY2olig condensates in experiments shown in (**f**).

To test DisCo with CRY2, we fused FRB at the N-or C-terminus of CRY2olig, along with EGFP or mCherry for tracking (e.g., EGFP-FRB-CRY2olig, CRY2olig-FRB-mCh), and used mCh-FKBP or mCh(K70N)-FKBP, respectively, as the C-BLOCK protein. We were curious whether recruitment of a C-BLOCK protein adjacent to CRY2olig, as in DisCo, rather than directly to CRY2, could result in disruption of its light-induced condensate state (Fig. 4a). In both configurations, light-induced condensates rapidly dissolved after rapamycin addition, even under conditions of constant blue light illumination (Fig. 4b, Supplementary Movie 3). More than 90% of the cytosolic condensates dissolved within 3 min (Fig. 4c, d), although nuclear clusters persisted for longer. Condensate dissolution required the FRB attached to CRY2olig, as condensates lacking this domain could not be disrupted (Fig. 4c). We also tested a version of CRY2olig, ‘CRY2oligC9’, containing nine residues (ARDPPDLDN) appended to the C-terminus, which was previously shown to reduce CRY2 colocalization with nuclear components^41^. Cells expressing this version, EGFP-FRB-CRY2oligC9 also formed condensates that rapidly dissolved upon rapamycin-induced recruitment of mCh-FKBP, with improved dissolution in the nucleus (Supplementary Fig. 4 and Supplementary Movie 4). After rapamycin addition, we could observe transient recruitment of mCh-FKBP into the condensates over ~1 min, coincident with condensate dissolution (Supplementary Movie 4).

With CRY2 and CRY2olig condensates, incubation in dark results in condensate dissolution and restoration of a uniform dilute state^5–7^. The kinetics of condensate dissolution mirrors the timing of CRY2 dark photoreversion, as light-excited CRY2 returns to a non-self-associating ground (dark) state. We found that while condensates of CRY2olig-FRB-mCh incubated in dark but without rapamycin had a similar dissolution rate as CRY2olig-mCh (tau = 472±59 s), the same condensates dissolved ~7 times faster (tau = 65±8 s) using DisCo (Fig. 4d). Importantly, condensates dissolved even in the presence of constant light that maintains CRY2olig in its light-activated state, indicating that DisCo induces disruption by a mechanism distinct from dark photoreversion. Consistent with prior results with CRY2olig-mCherry in FRAP experiments^8,13^, EGFP-FRB-CRY2olig condensates were poorly mobile, showing only ~20% recovery from photobleaching after 10 min (Fig. 4e). Taken together, our FRAP results with FUS, HTT, and CRY2olig condensates indicate that FRAP mobility, on its own, is not indicative of whether condensates are susceptible to be disrupted by DisCo.

We next examined whether an independent protein–protein interaction, rather than the rapamycin-based CID system, could be used for disruption. We replaced the FRB/FKBP pair with calmodulin (CaM) and calmodulin binding peptide M13 (CBP), which interact in the presence of Ca^2+^ ^42^, and used CRY2oligC9, with reduced nuclear binding. Cells expressing EGFP-CaM-CRY2oligC9 formed condensates upon blue light illumination. The addition of ionomycin (to raise intracellular Ca^2+^) triggered the recruitment of mCh-CBP to the scaffold protein, followed by condensate disruption on the same time scale as with FRB/FKBP/rapamycin (tau = 22±2 s) (Fig. 4f, g). Condensates exposed to ionomycin in the absence of C-BLOCK (no mCh-CBP) were not disrupted (Fig. 4f, right). To examine reversibility, we added the Ca^2+^ chelator EGTA to lower cellular Ca^2+^ levels, which resulted in reformation of condensates (Supplementary Fig. 5). These results show that, as demonstrated with zapalog, rounds of condensate formation and dissolution can be induced using DisCo.

### CRY2 homo- and hetero-interactions can be independently controlled using DisCo

**Fig. 5:**
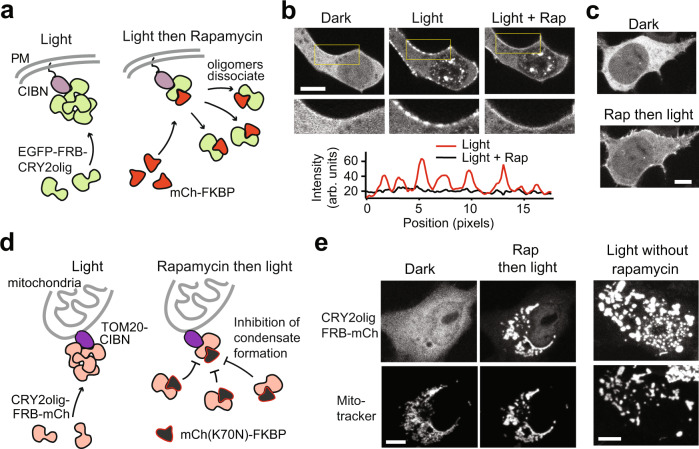
DisCo blocks CRY2olig self-interaction while maintaining other protein–protein interactions. **a** Schematic of plasma membrane recruitment experiments, testing effect of DisCo on ability of CRY2 to interact with a plasma membrane anchored CIBN. **b** HEK293T cells expressing CIBN-CaaX, EGFP-FRB-CRY2olig, and mCh-FKBP as C-BLOCK were illuminated with light (488 nm, 200 ms pulse every 20 s) 18–22 h post-transfection. Light-induced CRY2olig condensate formation and recruitment of condensates to the plasma membrane through CIBN interaction. The addition of 333 nM rapamycin (3 min after light onset, ‘Light+Rap’) disrupted condensates but maintained CRY2olig-CIBN interaction. Magnified images in the second row correspond to the areas outlined in yellow. Graph below shows intensity profile of fluorescence at the plasma membrane from the magnified areas, indicating the loss of clustering with rapamycin addition. The experiment was repeated a second time with similar results. Scale bar, 10 µm. **c** C-BLOCK recruitment prior to light application prevents CRY2olig condensate formation, while allowing CRY2-CIBN association. Experiment was performed as in (**b**), but with rapamycin added 25 min prior to illuminating cells with light (488 nm, 200 ms pulse every 20 s for 8 min). The experiment was repeated a second time with similar results. Scale bar, 10 µm. **d** Schematic of mitochondrial recruitment experiments, testing effect of DisCo on interaction of CRY2 with CIBN anchored at the mitochondria. **e** COS-7 cells coexpressing TOM20-CIBN, CRY2olig-FRB-mCh and mCh(K70N)-FKBP as C-BLOCK were imaged in dark (‘Dark’), then treated with 333 nM rapamycin followed by light (488 nm, 100 ms every 30 s) (“Rap then light”). CRY2olig treated with rapamycin prior to light does not form condensates but is still recruited to CIBN. At far left are images of cells illuminated with light in the absence of rapamycin, which results in CRY2olig oligomerization at the mitochondrial membrane and mitochondrial compaction. The experiment was repeated a second time with similar results. All scale bars, 10 µm.

In addition to undergoing homo-oligomerization, CRY2 interacts with other proteins, including CIB1, an interaction that has been extensively used for optogenetic applications^43^. Normally, both homo (CRY2-CRY2) and hetero (CRY2-CIB1) interactions are triggered by light, making it impossible to gain independent control over them. We examined whether DisCo could be used to specifically block CRY2-CRY2 interactions while maintaining CRY2-CIB1 interactions. We used CRY2olig in these studies rather than wild-type CRY2, as this variant forms visible, trackable condensates within cells, and CIBN, a truncated 1-170 residue version of CIB1^43^. We localized CIBN to the plasma membrane (CIBN-CAAX) and coexpressed with EGFP-FRB-CRY2olig and C-BLOCK (mCh-FKBP) in HEK293T cells (Fig. 5a). CRY2olig condensates formed with light and colocalized with CIBN at the plasma membrane. Addition of rapamycin disrupted CRY2olig condensates, but CRY2olig remained bound to CIBN at the plasma membrane (Fig. 5b). Addition of rapamycin prior to light addition prevented condensate formation, but CRY2olig was still recruited to CIBN at the PM (Fig. 5c).

We extended this work in COS-7 cells, testing recruitment of CRY2olig-FRB-mCh to mitochondrial-localized CIBN (TOM20-CIBN) (Fig. 5d). Cells expressing CRY2olig-FRB-mCh and C-BLOCK (mCh(K70N)-FKBP) but lacking rapamycin showed light-induced clustering of CRY2olig-FRB-mCh and recruitment to the mitochondria; however, this resulted in mitochondrial compaction, possibly due to CRY2-CRY2 interaction on neighboring mitochondria promoting fusion (Fig. 5e, “Light without rapamycin”). In contrast, in cells treated with rapamycin prior to illumination, CRY2olig-FRB-mCh was recruited to mitochondria but did not form large clusters (Fig. 5e, “Rap then light”). Compaction of mitochondria within rapamycin-treated cells was substantially reduced compared to untreated cells. Our studies indicate that DisCo can be used with light, CRY2, and CIBN to achieve control over three different protein states in the same cell: non-clustered, uniform CRY2 protein (in dark), CRY2 clusters that colocalize with CIBN (in light), and CRY2 in non-clustered form that colocalizes with CIBN (in light with rapamycin).

### Use of CRY2olig and DisCo to inducibly and reversibly manipulate protein function

Many natural proteins have their activities regulated by oligomerization, and studies have demonstrated the use of CRY2 and CRY2olig to synthetically regulate such activity states with light. For example, CRY2 has been used to modulate lipid signaling and cell cycle^7^, induce Raf1 kinase activity^16,17^, modulate signal transduction in T cells^18^, and to induce condensates of proteins associated with neuropathologies^12–14^. While condensate-dependent functions can be rapidly induced with CRY2, there is no way to rapidly revert this process. We tested whether we could use DisCo to inducibly reverse activities dependent on CRY2 homo-oligomeric clustering, using clathrin-dependent endocytosis as a test case.

**Fig. 6:**
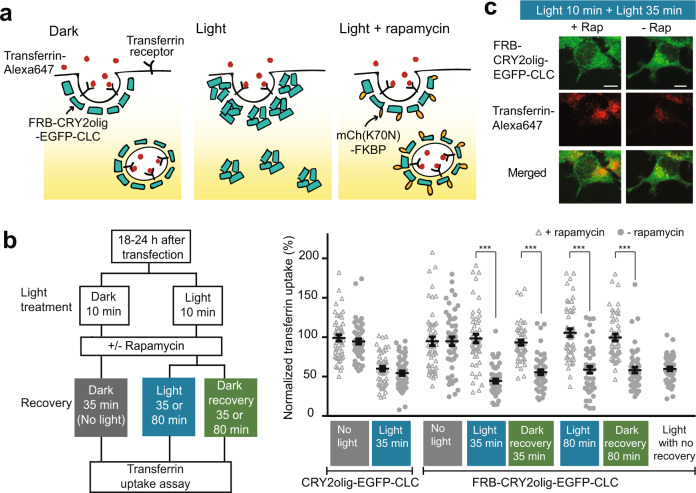
Use of DisCo to rapidly reverse endocytosis blockage induced by CRY2olig and light. **a** Schematic showing use of DisCo to reverse light-dependent blockage of clathrin-mediated endocytosis induced by CRY2olig clustering. **b** Timecourse (left) and results (right) of endocytosis assay. CRY2olig-clathrin light chain (CLC) constructs with or without a FRB hook were coexpressed in HEK293T cells along with mCh(K70N)-FKBP as C-BLOCK. 18–24 after transfection, cells were kept in dark (‘No Light’) or exposed to blue light (465 nm, 1 s pulse every 30 s for 10 min). Cells were treated with 333 nM rapamycin or vehicle, then incubated for 35 or 80 additional minutes in light (465 nm,1 s pulse every 30 s) (“Light”) or dark (“Dark recovery”) as indicated, then assayed for transferrin uptake. As a control for the reduction of transferrin uptake due to light treatment, cells were treated with light (465 nm, 1 s pulse every 30 s for 10 min) and assayed for transferrin uptake immediately after the light treatment (Light with no recovery). Transferrin uptake is expressed as a percent of results obtained from neighboring untransfected cells (set at 100%). Data represents average and error (s.e.m., *n* = 50 cells). Parametric t-test (two-tailed) between + /- rapamycin-treated cells showed a significant difference (***) with *p* < 0.0001 (Light 35 min, *p* = 1.4 ×10^−14^; Dark recovery 35 min, *p* = 1.4×10^−14^; Light 80 min, *p* = 1.7×10^−10^; Dark recovery 80 min, *p* = 5.5 ×10^−11^).The experiment was performed an additional time with similar results. **c** Representative images of transferrin uptake in experiment shown in (**b**). Cells treated with DisCo (“+ Rap”) show recovery from CRY2olig-CLC clustering and normal uptake of transferrin even when incubated in light. The experiment was performed an additional time with similar results. Scale bars, 10 µm.

In previous work, it was demonstrated that clathrin-dependent endocytosis could be blocked by CRY2olig homo-oligomerization^5^. Cells overexpressing a fusion of CRY2olig to clathrin light chain (CRY2olig-mCh-CLC) showed a ~60% reduction in uptake of transferrin, mediated through clathrin-mediated endocytosis, upon light-induced clustering^5^. We attached a FRB hook to CRY2olig to generate FRB-CRY2olig-EGFP-CLC, and coexpressed this in HEK293T cells along with mCh(K70N)-FKBP as C-BLOCK (Fig. 6a). Cells expressing FRB-CRY2olig-EGFP-CLC formed clusters in light and showed a ~50% reduction in transferrin uptake, similar as previously observed with CRY2olig-mCh-CLC (Fig. 6b, c). Cells incubated in dark with no rapamycin after a 10 min light treatment (Dark recovery) showed only minor recovery after 35 or 80 min, to 56% or 65% respectively. Meanwhile, cells treated with rapamycin after the initial light treatment showed full recovery after 35 min, even under constant light illumination. The rapamycin-induced response required FRB-mediated recruitment, as control cells coexpressing CRY2olig-EGFP-CLC (with no FRB) and mCh(K70N)-FKBP showed no rapamycin-dependent recovery (Fig. 6b). These results demonstrate the utility of using DisCo to extend the applications of light-induced clustering tools, allowing researchers to rapidly form or dissolve condensates using orthogonal inducible inputs.

## Discussion

In this report, we describe a generalizable method, DisCo, that uses inducible dimerizers to dissolve protein condensates at specific times within cells. DisCo provides a modular, inducible, and targeted chemical approach to dissolve and prevent condensate formation, with advantages over other approaches. Other chemical approaches such as use of 1,6-hexanediol^28^ can inducibly disrupt a wide range of condensates; however, this chemical acts generally on all condensates and lacks specificity. Natural and synthetic proteins have been identified that interact with and disrupt specific condensates, such as profilin or intrabodies (which disrupt condensates of huntingtin Exon 1), or β-karyopherin (which disrupts FUS condensates); however, while such approaches provide specificity, they are not inducible or generalizable for use with other proteins. Light-dependent approaches have been developed in which synthetic condensates are disrupted by light^30^; however, the condensates formed through these methods differ significantly from native protein condensates. With DisCo, only a small, 11 kDa FRB hook is attached to the native protein, causing relatively minimal perturbation.

While protein dimerization tools have previously been used to induce condensates and protein assemblies^8–10,44^, to our knowledge this work represents a unique use of inducible protein dimerization and recruitment to block or break up other ordered protein assemblies. In addition to validating our approach with a rapamycin-based CID system, we demonstrate with the photocleavable CID zapalog that the DisCo approach is reversible and can be used to cycle between free diffusing and condensate states of FUS. We also demonstrate the approach is sufficiently versatile to achieve disruption using a completely different protein interaction pair, such as we demonstrate with the Ca2+-dependent interaction between CaM and a CaM binding peptide, and using different C-BLOCK proteins (mCherry-FKBP or FKBP alone). In addition to demonstrating the approach with different induced binders, we demonstrate use with different scaffold molecules that drive condensate formation, including those formed by LCRs such as FUS, as well as those formed by oligomeric proteins such as CRY2olig. Our work showing success using DisCo with different induced binders to disrupt different target protein scaffolds suggests the approach is likely to be generally applicable.

With CRY2olig, we demonstrate the ability to use DisCo, CRY2, CIBN, and light to switch between three different CRY2/CIBN conditions: condensate/recruited, non-condensate/recruited, and non-condensate/not recruited (Fig. 5). We also demonstrate the ability to use DisCo to rapidly reverse phenotypes induced by CRY2 light-dependent clustering. CRY2 clustering-based approaches are widely used for optogenetic manipulation of cell processes and the DisCo approach can be easily implemented to provide a light-independent toggle to induce fast dissolution of clustered protein. Using clathrin-mediated endocytosis as a readout (Fig. 6), we demonstrate how DisCo can be combined with CRY2olig, using CRY2olig to block a process with light through light-mediated clustering, then using DisCo to rapidly revert the blocked state, speeding up the recovery of clathrin-mediated endocytosis. While clustered CRY2 and CRY2olig returns to a soluble form over time in dark, in some cases the time frame for recovery is extended, as demonstrated with FRB-CRY2olig-EGFP-CLC that showed minimal recovery even after 80 min in dark. Due to its rapid action, we expect the DisCo approach will be particularly useful for studies using CRY2 to control signaling and other cellular processes that occur on rapid time-scales^45^.

In studies with FUS, we find that recruitment of a monomeric protein to a region outside of the LCR that drives the condensate cohesion is sufficient to trigger condensate dissolution. Given that biomolecular condensates are proposed to play an important role in a number of neurodegenerative diseases, a growing area of therapeutics is focused on approaches to block or disrupt disease-associated condensates. Many of these efforts have focused on identifying chemicals or proteins that bind condensate-forming proteins directly at the sites involved in condensate formation (for example, the LCR of FUS). Our work demonstrates that condensates can be effectively disrupted by binding at a region of the protein adjacent to the region forming the condensate, providing new opportunities for therapeutic treatment. We envision that the DisCo approach could be used to test the effect of condensate disruption at specific times in a variety of condensate-related pathologies, and to explore treatments to maintain protein in a uniform phase state.

While our results convincingly demonstrate the use of a recruited ligand to disrupt condensates, the precise mechanism for disruption is not established. One compelling model relates to the concept of polyphasic linkage^46^, where preferential binding of a ligand to the dilute phase of a phase-separated protein can shift the saturation concentration of a phase transition. In a recent computational modeling study, Ruff et al.^27^ used polyphasic linkage theory to demonstrate how preferential binding of a monovalent ligand to a ‘spacer’ region of a scaffold (separate from the “sticky” condensate-forming region) in the dilute phase state could result in condensate destabilization. Applying these concepts in our DisCo experiments with FUS, the binding of monovalent ligand (C-BLOCK) would be predicted to increase the effective solvation volume of the spacer (FRB hook), lead to decreased cooperativity between interactions driving the condensate-forming behavior (FUS IDR), and effectively increase the concentration at which the scaffold protein forms a phase-separated state. In the Ruff et al. study, binding of a monovalent ligand to the spacer or sticker site was predicted to disrupt the condensate, while binding of a multivalent ligand to the spacer was predicted to enhance phase separation due to the additional crosslinking interactions^27^, consistent with our findings recruiting monovalent mCherry-FKBP and FKBP alone, compared to the divalent mCherry-FKBP-FKBP. While the polyphasic linkage concept provides a possible explanation for how recruiting a monovalent ligand to a protein at sites not directly related to the interactions that drive the phase transition can lead to condensate destabilization, there are other explanations for the observed results. Indeed, given the differences between the liquid-like condensates formed by FUS and the more solid-like clusters formed by CRY2olig, different mechanisms could be at play. Homo-oligomerization of CRY2olig, for example, is sensitive to steric factors^17^, and recruitment of ligands at specific sites could destabilize oligomeric interfaces or compete with oligomeric assembly. Despite the current unknown mechanisms, we expect that DisCo will become a valuable platform to further test different models of ligand binding in condensates, and to explore how different ligand parameters such as affinity, valency, amino acid composition, and charge can tune condensate dynamics.

In summary, our work provides a generally applicable method to rapidly disrupt functional condensates within cells. The ability to rapidly and reversibly control the induction and turnoff of disease-related or synthetic condensates will enable a better understanding of the consequences of condensate formation within cells. We envision that DisCo will enable future experiments to understand the role of ligands as regulators of multicomponent condensates. These tools can provide a platform to probe outstanding questions in condensate biology, such as how long after a condensate is formed is a biological response elicited, or probing the effects of dissolving or preventing the formation of condensates during stress states. Combining DisCo with existing strategies to form disease-relevant condensates will provide powerful tools to probe the consequences of condensate formation and disruption at different disease stages and times in model organisms.

## Methods

### Plasmid construction

A full description of the sequences, cloning method, and primers used for each construct are provided in Supplementary Data 1. We thank Dr. Nicolas Fawzi, Dr. Takanari Inoue, and Dr. Douglas Kim for providing constructs (via Addgene) used in plasmid construction including FUS (Addgene #98651)^47^, FRB containing T2098L (Addgene #103776)^9^, FKBP (Addgene #103777)^9^, CaM and CBP (Addgene #40753)^48^. We also thank Dr. Robert Hughes for providing Q72HTT-GFP and Dr. Matt Kennedy for providing the template for amplification of ecDHFR.

All PCR reactions used Phusion polymerase (New England Biolabs). Cloning was carried out using DNA digestion and ligation with T4 DNA Ligase or Quick Ligase (NEB), or using Gibson assembly. Gibson assembly reactions were performed by incubating 200 ng of the insert DNA and 200 ng of the linearized vector at 50˚C for 30 min in a homemade Master Mix (0.1 M Tris, 10 mM MgCl_2_, 0.2 mM dNTPs, 10 mM DTT, 50 mg/ml PEG-8000, 1 mM NAD, 0.8 U T5 exonuclease (NEB), 0.5 U Phusion polymerase (NEB), 80 U Taq Ligase (MC Labs)).

### Cell culture

HEK293T or COS-7 cells were cultured in Dulbecco’s modified Eagle medium (DMEM) (Corning) supplemented with 10% fetal bovine serum (FBS) (Sigma) and 1x Penicillin-Streptomycin (Corning) at 37 ° C with 5% CO_2_. HEK293T cells were transfected using calcium phosphate or Lipofectamine 2000 (Invitrogen), according to the manufacturer’s protocol. All COS-7 cells were transfected using Lipofectamine 2000. For CRY2olig studies, samples were wrapped in aluminum foil immediately after transfection and kept in dark until the following day. Subsequent sample manipulations were carried out using a red safelight. Light-treated cells were illuminated using a custom programmable LED light source^49^ (465 nm, 1.1 mW/cm^2^). A 1 s light pulse was delivered every 30 s (3.3% duty cycle) for all experiments, unless differently indicated.

### Live cell imaging experiments

Cells were seeded onto 35-mm glass bottom culture dishes. The following day, for HEK293T cells, calcium phosphate transfection methods were used to transfect 500 ng of each specified FUS, Q72HTT, or CRY2olig plasmid DNA, and 1000 ng of each C-BLOCK protein. Unless specified otherwise, cells were moved to HBSS imaging buffer (Hanks Balanced Salt Solution, 1.26 mM CaCl_2_, 0.41 mM MgSO_4_, .49 mM MgCl_2_, 5.33 mM KCL, 138 mM NaCl, 0.44 mM KH_2_PO_4_, 0.34 mM Na_2_HPO_4_, 4.17 mM NaHCO_3_, 5.56 mM D-glucose, and 20 mM HEPES) for live cell imaging 18 to 22 h after transfection. Where specified, 333 nM rapamycin was added directly to the samples during live cell imaging.

To examine the effect of DisCo over arsenite-induced condensates of FUS, COS-7 cells were transfected with 250 ng of EGFP-FRB-FUS-FRB or EGFP-FRB-FUS and 500 ng of mCh-FKBP plasmid. 18–22 h after transfection, cells were imaged during treatment with 1 mM sodium arsenite. After arsenite-induced formation of FUS condensates, 333 nM rapamycin was added.

To examine recruitment of CRY2olig to mitochondria, COS-7 cells were transfected with 500 ng of each specified plasmid DNA. 18–22 h after transfection, cells were moved to imaging buffer containing 100 nM Far-Red MitoTracker (Invitrogen). For zapalog experiments, HEK293T cells were transfected with 500 ng EGFP-DHFR-FUS and 500 ng mCh-FKBP. 4 h after transfection, media was replaced and 500 nM zapalog was added. Cells were subsequently kept in the dark. 18–22 h after transfection, cells were moved to imaging buffer containing 500 nM zapalog. For experiments involving CaM/CBP cells were moved to PBS prior to live cell imaging. 2.5 mM CaCl_2_ and 3 µM ionomycin were added directly to samples during imaging to increase intracellular calcium. To reverse the Ca^2+^ increase, 10 mM EGTA was added.

During imaging, samples were kept at 33.5˚C in a light-tight stage top incubator enclosure. Cells were imaged using either of three systems: (1) an Andor Dragonfly 301 spinning disc imaging system with Olympus IX73 base and four-line ILE laser merge module and controller. Images were taken using a 60x UplanSApo 1.35 NA oil objective and collected on a 1024 ×1024 pixel Andor iXon EM-CCD camera. Data was acquired using Fusion 2.0 or iQ3 (Andor). (2) A Zeiss AxioObserver Z1 inverted microscope equipped with a Yokogawa CSU-X1 spinning disk unit, a 63x/NA 1.4 oil immersion objective, a mSAC unit for correction of spherical ablation (3i), a Vector live specimen scanner (3i), a mSwitcher ms optical switching unit (3i) and a Photometrics 512×512 EM-CCD camera. Slidebook 6 was used for all collection of images on this system. 3) Olympus IX71 microscope equipped with a spinning disc scan head (Yokogawa) and a 60x/NA 1.4 oil immersion objective. Excitation illumination was delivered from an AOTF controlled laser launch (Andor Technology) and images collected on a 1024 ×1024 pixel EM-CCD camera (iXon; Andor Technology). After initial image collection, ImageJ 1.46r was used for all image analysis.

### Rapalog treatment

For extended treatment with chemical dimerizer, we used the rapalog AP21967, iDimerize Inducible A/C Heterodimerizer (Takara Bio). HEK293T cells were seeded onto coverslips in 12-well plates. 500 ng of each specified FUS or 1000 ng for Q72HTT plasmid DNA, and 1 µg of C-BLOCK DNA was transfected (calcium phosphate method) into HEK293T cells. After 4 h, medium was replaced with fresh medium with 500 nM AP21967. After 18 h for FUS samples and 44 h for Q72HTT samples, coverslips were washed in phosphate buffered saline (PBS), fixed in 4% paraformaldehyde (Sigma), and mounted on a glass slide with Fluoromount-G (Southern Biotech). For FUS and Q72HTT constructs, fixed slides were imaged using the Andor Dragonfly confocal system.

### Image analysis

All images were analyzed using ImageJ/Fiji software. For all experiments, measurements and quantification were from distinct samples (no repeat samples). Initial Z-stacks were first compiled into a maximal projection. Signal within the condensate was specified by thresholding (Otsu’s or MaxEntropy method) in ImageJ. To calculate the fluorescence intensity in the condensates for both the initial (pre rapamycin) as well as the final (post rapamycin) images, we quantified the mean signal intensity within the condensate (dense phase) and subtracted the mean signal intensity in the cytosol (dilute phase), then multiplied by the condensate area, e.g., (Area_dense_)(MeanIntensity_dense_-MeanIntensity_dilute_). This value was then normalized to the total cell integrated density to give a value representing the fraction of total signal within the condensate, (Fraction_pre_, prior to rapamycin addition, and Fraction_post_, final image after rapamycin). We defined the % disruption efficiency as equal to the difference of these values, divided by the pre rapamycin value (100*(Fraction_pre_-Fraction_post_)/Fraction_pre_)).

To carry out the correlation in Figs. 1f, 3a, and Supplementary Fig. 1d, for each cell we plotted the % disruption efficiency on the X-axis and the ratio of the background-subtracted mean signal intensity within the condensate divided by the mean signal intensity out of the condensate e.g., (MeanI_dense_/MeanI_dilute_). To calculate the condensate intensity ratio, we measured the integrated intensity within condensates in the maximum projection images, and divided by the total cell integrated density (background subtracted). Inhibition of FUS and Q72HTT condensate formation with or without rapalog was quantified as the total intensity of GFP in condensates (MaxEntropy threshold method) normalized to the total integrated density of the cells (Mean threshold method). For each sample 6 confocal images were merged to have a final area of 1.4 mm×1.4 mm.

To calculate the kinetics of condensate dissociation, the fluorescence intensity within condensates (Otsu’s thresholding method) was plotted as a function of time. For FUS, experimental data were fitted to a sigmoid function using Igor Pro 5. The characteristic times (tau) were calculated from the sigmoidal fits. For CRY2olig constructs, experimental data that correspond to time points after rapamycin addition were fitted to an exponential function using Igor Pro 5. The characteristic times (tau) were calculated from sigmoidal fits.

### Statistical comparison

Significance for experiments comparing populations was determined using GraphPad Prism6 software. A non-parametric one-way ANOVA test was used for Fig. 1h and Supplementary Fig. 1b, a Kolmogorov–Smirnov (two-tailed) test was used for Fig. 1c, and a parametric t-test (two-tailed) was used for Fig. 6b.

### FRAP experiments

Photobleaching experiments were performed at 33.5 °C, using either the 3i Marianas spinning disk confocal, or the Olympus IX71 with Yokogawa spinning disc scan head described previously. For the Olympus system, laser illumination for FRAP experiments was delivered using galvometric laser-scanning mirrors (FRAPPA; Andor Technology). For both instruments, 50% laser power with a 1–2 ms dwell time was used. Photobleaching pulses were calibrated so that they bleached no more than 60–85% of the original signal. To generate Figs. 1i, 3b, and 4e containing FRAP analysis, each photobleached region in the condensate was selected and the background free integrated density at each time point was measured. To account for global photobleaching effects, the integrated density of the bleached region was normalized to the background free total cell integrated density. The normalized values were mapped to an [0,1] interval, setting the zero value as the normalized integrated density at the photobleaching time point and the 1 value as the average of the normalized integrated density before photobleaching.

### Endocytosis studies

HEK293T cells were seeded onto 18-mm coverslips in a 24-well plate and transfected with 750 ng of FRB-CRY2olig–EGFP-CLC or CRY2olig-EGFP-CLC (control) and 1000 ng mCh(K70N)-FKBP using calcium phosphate transfection. Samples were incubated in the dark for 18–24 h, then moved to serum-free media 20 min before initiating light treatment. Cells were maintained in the dark or treated with blue light for 10 min (1 s pulse, every 30 s, 465 nm, 1.1 mW cm^−2^), then 333 nM rapamycin was added and light or dark treatment was maintained for an additional 35 or 80 min. To measure transferrin uptake, cells were incubated with 50 µg/ml transferrin-AlexaFluor^®^ 647 (Life Technologies) for 10 min, then washed in PBS, fixed in 4% paraformaldehyde, and mounted on a glass slide. Cells were imaged on the Andor Dragonfly system described above. Cells showing medium to high expression (top 25–100% highest GFP fluorescence) were chosen for analysis, with 50 cells quantified for each experimental condition. For each condition, the background-subtracted fluorescence intensity of transferrin-AlexaFluor^®^ 647 in transfected cells was normalized and expressed as a percent of the transferrin-AlexaFluor^®^ 647 signal in untransfected cells.

### Reporting summary

Further information on research design is available in the Nature Research Reporting Summary linked to this article.

## Supplementary information

Supplementary Information

Description of Additional Supplementary Files

Supplementary Data 1

Supplementary Movie 1

Supplementary Movie 2

Supplementary Movie 3

Supplementary Movie 4

Reporting Summary

## Data Availability

All data generated and analyzed for this study are included in the article and the supporting information. Additional raw data (images, etc) is available from the authors upon reasonable request. Source data are provided with this paper.

## Source data

Source Data
