# Supplementary material for: A modular tool to query and inducibly disrupt biomolecular condensates
Source: Nat Commun. 2021 Mar 22;12:1809. doi: 10.1038/s41467-021-22096-1 (PMC7985322; doi:10.1038/s41467-021-22096-1)
Supplement: Supplementary file 1 — Supplementary Information [file 41467_2021_22096_MOESM1_ESM.pdf]

Supplementary information

A modular tool to query and inducibly disrupt biomolecular condensates

Carmen N. Hernández-Candia[1], Sarah Pearce[1], and Chandra L. Tucker[1,*]

[1]Department of Pharmacology, University of Colorado School of Medicine, Aurora, CO 80045, USA

*Correspondence: chandra.tucker@cuanschutz.edu

Supplementary Figure 1-5

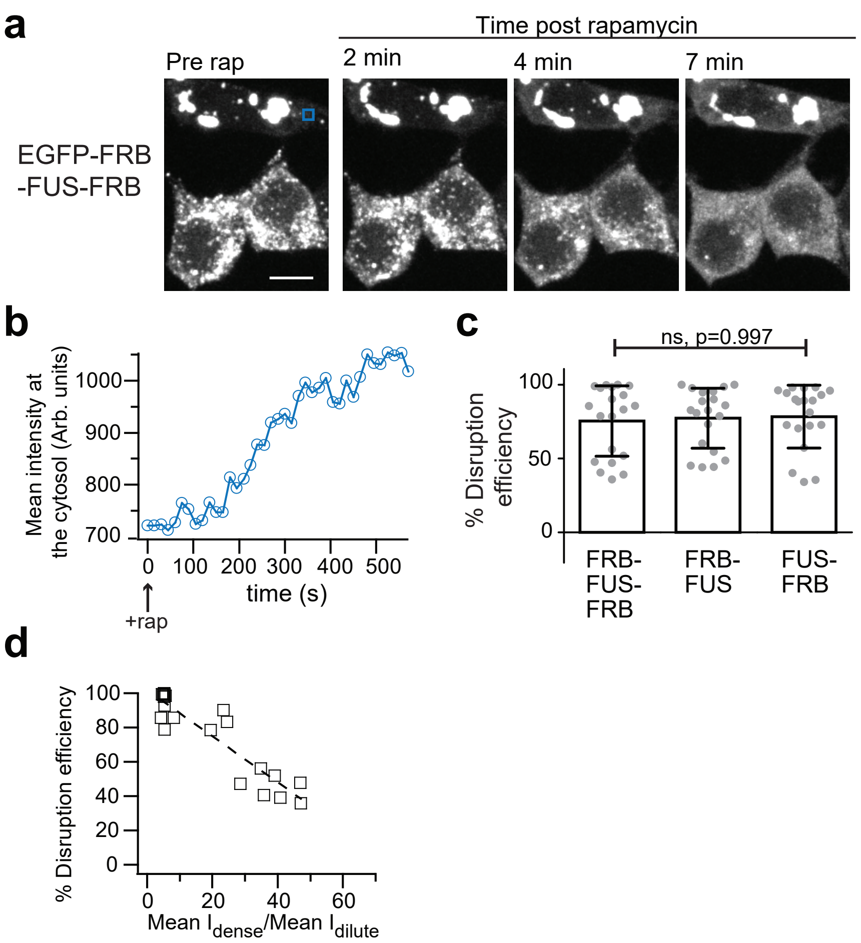

Supplementary Figure 1. DisCo using a non-fluorescent version of mcherry (K70N) as C-BLOCK shows similar results as mCh-FKBP. **a)** HEK293T cells coexpressing EGFP-FRB-FUS-FRB(a) or EGFP-FUS-FRB (b) and mCh(K70N)-FKBP form condensates that were dissolved after adding 333 nM rapamycin. The experiment was repeated 3 times with similar results. Scale bar, 10 µm. **b)** After adding rapamycin the GFP mean intensity at the indicated region of the cytosol (blue box in **a)** showed an increase. **c)** Placement of the FRB 'hook' at different orientations did not affect the % disruption efficiency. Cells co-expressing FUS with different orientations of FRB and mCh(K70N)-FKBP as C-BLOCK were treated with rapamycin to induce condensate dissolution. Data show average and error (s.d., n=20 cells examined over 3 independent experiments, ns, not significant, non-parametric one-way ANOVA test, p=0.997). **d)** Graph showing correlation between % disruption efficiency and the ratio of signal in dense vs dilute phase (Mean I_{dense})/(Mean I_{dilute}) from the initial (pre-rapamycin) image.

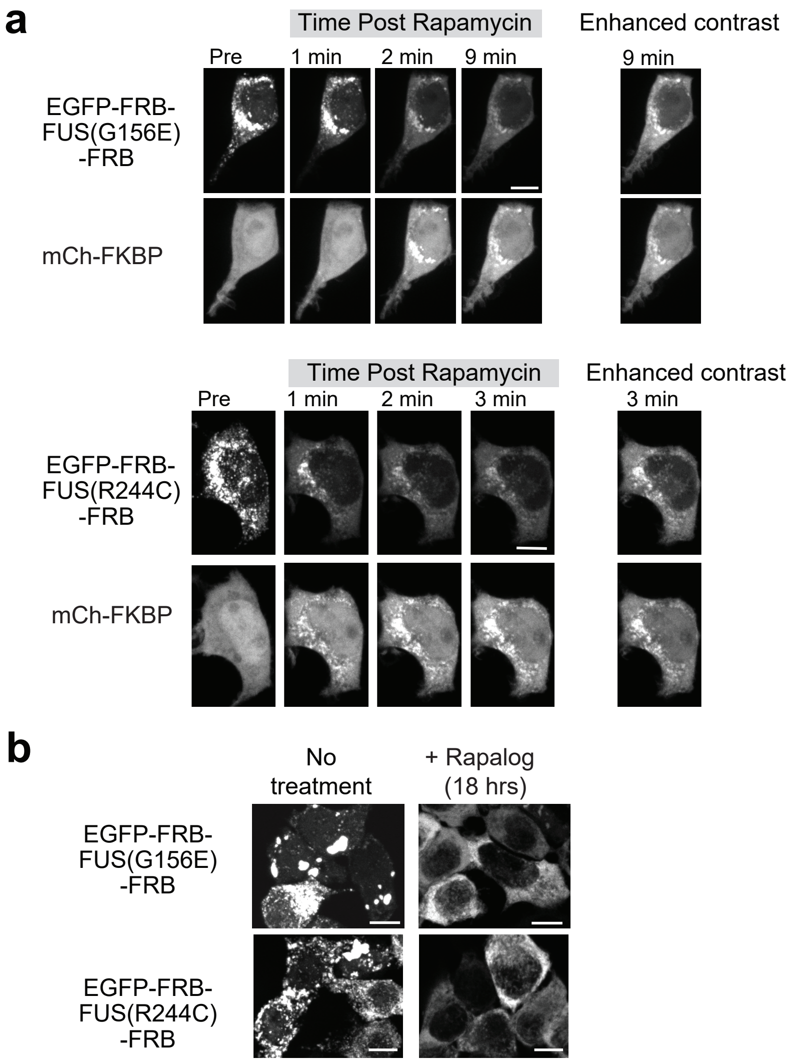

Supplementary Figure 2. DisCo approach with representative FUS variants associated with ALS disease. **a)** Condensates formed by FUS variants were disrupted by rapamycin/C-BLOCK treatment. Cells coexpressing EGFP-FRB-FUS(G156E)-FRB or EGFP-FRB-FUS(R244C)-FRB and mCh-FKBP were treated with 333 nM rapamycin 18-22 hrs after transfection. The last figure panel is reproduced at right with enhanced contrast to clearly show the remaining FUS clusters and colocalization with mCh-FKBP. The experiment was repeated three times with similar results. Scale bar, 10 µm. **b)** Prior treatment of cells expressing FUS mutant proteins prevents formation of condensates. Cells coexpressing EGFP-FRBFUS(G156E)-FRB or EGFP-FRB-FUS(R244C)- FRB and mCh-FKBP were treated with 500 nM AP21967 (rapalog) for 18 hrs, added 4 hrs after transfection, and imaged at 22 hrs post transfection. The experiment was repeated three times with similar results, with representative images from a single experiment shown. Scale bar, 10 µm.

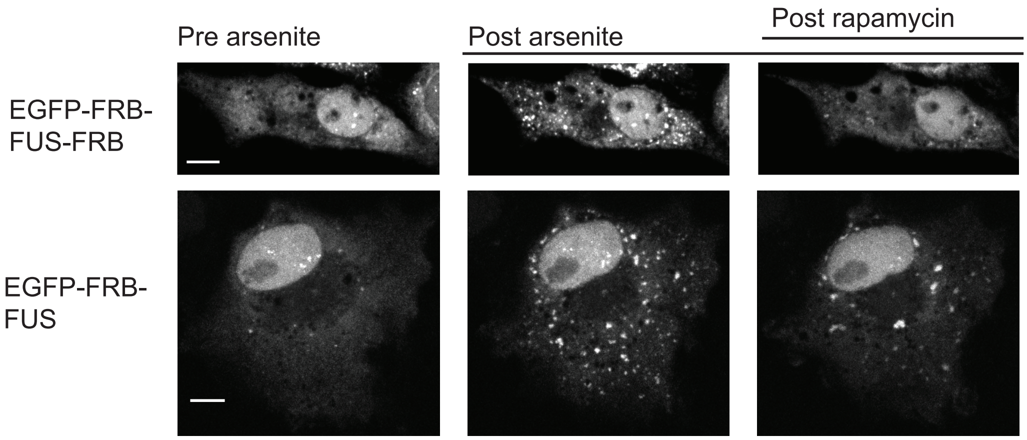

Supplementary Figure 3. DisCo approach can be used to dissolve arsenite-induced condensates of FUS. Cos-7 cells with a low expression of EGFP-FRB-FUS-FRB or EGFP-FRB-FUS and mCh-FKBP were treated with 1 mM sodium arsenite to induce condensate formation. After condensates were formed cells were treated with 333 nM rapamycin to induce the dissolution of the condensates. Representative images shown; the experiment was repeated a second time with similar results. Scale bars, 10 μm.

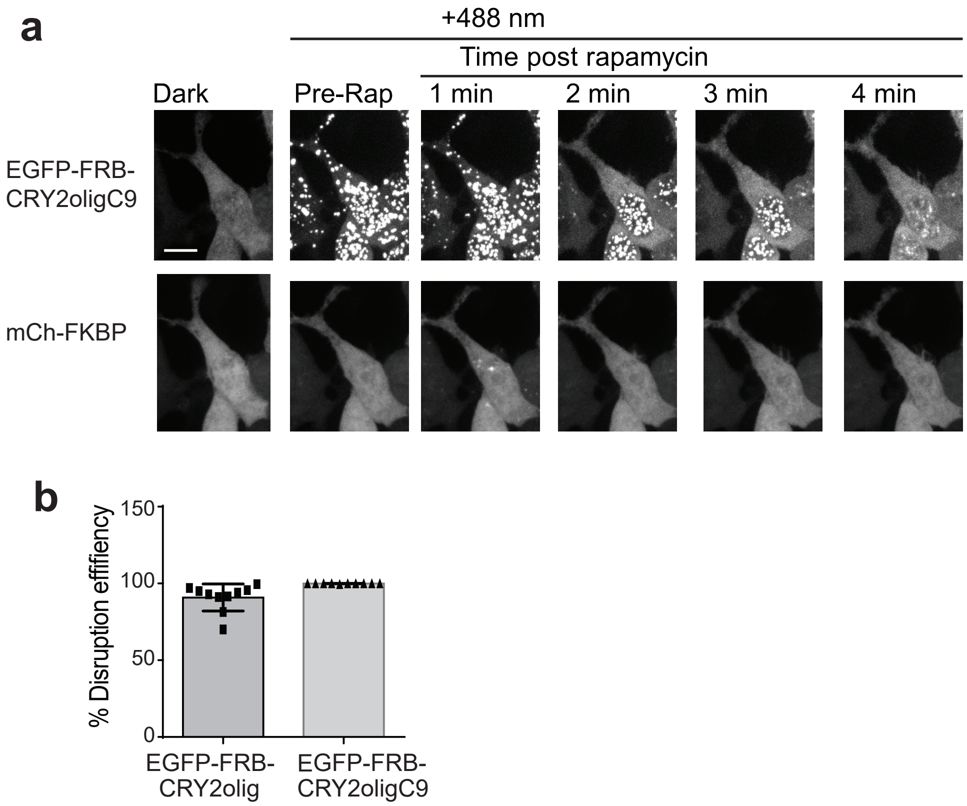

Supplementary Figure 4. Use of DisCo with CRY2oligC9. **a)** Representative images of HEK293T cells coexpressing EGFP-FRB-CRY2oligC9 and mCh-FKBP form cytosolic and nuclear condensates that are completely dissolved after rapamycin addition. The experiment was repeated 3 time with similar results. Scale bar, 10 μm. **b)** Comparison of % disruption efficiency of DisCo with CRY2olig vs. CRY2oligC9. The % disruption efficiency (quantified as in Figure 1c) was determined for cells coexpressing EGFP-FRB-CRY2oligC9 or EGFP-FRB-CRY2olig and mCh-FKBP as C-BLOCK. Data shows average and error (s.d., n=10 cells examined over 3 independent experiments). For **(a)** and **(b)**, cells were illuminated throughout the experiment (488 nm, 100 ms every 30 s), and 333 nM rapamycin was added 5 min after light onset.

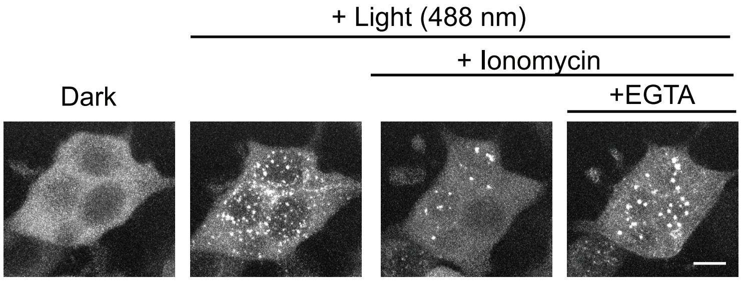

Supplementary Figure 5. Reversibility of Ca^{2+}-dependent DisCo. HEK293T cells coexpressing EGFP-Calmodulin-CRY2oligC9 and mCh-CBP were treated with light for 5 min (488 nm, 100 ms every 30 s) to induce condensates. Then 2.5 mM $CaCl_2$ and 3 μM ionomycin were added to induce recruitment of mCh-CBP 5 min after light onset. The Ca^{2+}-induced effect was reverted by adding the chelator EGTA (10 mM) 15 min after light onset. The experiment was repeated a second time with similar results. Scale bar, 10 μm
