# Supplementary material for: A modular tool to query and inducibly disrupt biomolecular condensates
Source: Nat Commun. 2021 Mar 22;12:1809. doi: 10.1038/s41467-021-22096-1 (PMC7985322; doi:10.1038/s41467-021-22096-1)
Supplement: Supplementary file 2 — Description of Additional Supplementary Files [file 41467_2021_22096_MOESM2_ESM.pdf]

Description of Additional Supplementary Files

Supplementary Data 1. List of plasmid constructs and primers used in this study.

Supplementary Movie 1. Use of DisCo to disrupt FUS condensates. Shown are HEK293T cells expressing EGFP-FRB-FUS-FRB and mCh-FKBP. 333 nM rapamycin was added at t=0 s. Video shows EGFP (left) and mCh (right) fluorescence channels.

Supplementary Movie 2. Use of DisCo to disrupt FUS condensates. Shown are HEK293T cells expressing EGFP-FRB-FUS-FRB and mCh(K70N)-FKBP. 333 nM rapamycin was added at t=0s. Video shows EGFP fluorescence channel.

Supplementary Movie 3. Use of DisCo to disrupt CRY2olig condensates. Shown is a HEK293T cell expressing CRY2olig-FRB-mCh and mCh(K70N)-FKBP. Sample was illuminated with blue light throughout the experiment (488 nm, 100 ms every 30 s) to induce cluster formation. 333nM rapamycin was added 5 min after blue light onset. Video shows mCherry fluorescence channel.

Supplementary Movie 4. Use of DisCo to disrupt CRY2oligC9 condensates. Shown are HEK293Tcells expressing EGFP-FRB-CRY2oligC9 and mCh-FKBP. Cells were illuminated with blue light throughout the experiment (488 nm, 100 ms every 10 s) to induce cluster formation. After 2 min of blue light illumination, 333 nM rapamycin was added at t= 0 s. Video shows EGFP (left) and mCh (right) fluorescence channels.
