# Supplementary material for: A modular tool to query and inducibly disrupt biomolecular condensates
Source: Nat Commun. 2021 Mar 22;12:1809. doi: 10.1038/s41467-021-22096-1 (PMC7985322; doi:10.1038/s41467-021-22096-1)
Supplement: Supplementary file 8 — Reporting Summary [file 41467_2021_22096_MOESM8_ESM.pdf]

nature research

Corresponding author(s): Chandra L Tucker

Last updated by author(s): Feb 23, 2021

Reporting Summary

Nature Research wishes to improve the reproducibility of the work that we publish. This form provides structure for consistency and transparency in reporting. For further information on Nature Research policies, see our Editorial Policies and the Editorial Policy Checklist.

Statistics

For all statistical analyses, confirm that the following items are present in the figure legend, table legend, main text, or Methods section.

n/a	Confirmed	
☐	☒	The exact sample size (*n*) for each experimental group/condition, given as a discrete number and unit of measurement
☐	☒	A statement on whether measurements were taken from distinct samples or whether the same sample was measured repeatedly
☐	☒	The statistical test(s) used AND whether they are one- or two-sided<br>*Only common tests should be described solely by name; describe more complex techniques in the Methods section.*
☒	☐	A description of all covariates tested
☒	☐	A description of any assumptions or corrections, such as tests of normality and adjustment for multiple comparisons
☐	☒	A full description of the statistical parameters including central tendency (e.g. means) or other basic estimates (e.g. regression coefficient) AND variation (e.g. standard deviation) or associated estimates of uncertainty (e.g. confidence intervals)
☐	☒	For null hypothesis testing, the test statistic (e.g. *F*, *t*, *r*) with confidence intervals, effect sizes, degrees of freedom and *P* value noted<br>*Give P values as exact values whenever suitable.*
☒	☐	For Bayesian analysis, information on the choice of priors and Markov chain Monte Carlo settings
☒	☐	For hierarchical and complex designs, identification of the appropriate level for tests and full reporting of outcomes
☒	☐	Estimates of effect sizes (e.g. Cohen's *d*, Pearson's *r*), indicating how they were calculated

Our web collection on statistics for biologists contains articles on many of the points above.

Software and code

Policy information about availability of computer code

Data collection	Andor Fusion 2.0 imaging software, Andor IQ3.
Data analysis	ImageJ 1.46r, Igor Pro 5, GraphPad Prism6

For manuscripts utilizing custom algorithms or software that are central to the research but not yet described in published literature, software must be made available to editors and reviewers. We strongly encourage code deposition in a community repository (e.g. GitHub). See the Nature Research guidelines for submitting code & software for further information.

Data

Policy information about availability of data

All manuscripts must include a data availability statement. This statement should provide the following information, where applicable:

- Accession codes, unique identifiers, or web links for publicly available datasets
- A list of figures that have associated raw data
- A description of any restrictions on data availability

All data generated and analyzed for this study are included in the article and the supporting information. Source data is included with this publication. Additional raw data (images, etc) is available from the authors upon reasonable request.

Field-specific reporting

Life sciences study design

All studies must disclose on these points even when the disclosure is negative.

Sample size	Sample size was not chosen based on a statistical method. Sample sizes were chosen to ensure reproducibility of the data and estimate errors in measurement.
Data exclusions	During endocytosis studies, the cells expressing in the lowest quartile (lowest 25% expression) were not quantified (pre-established criteria) as to reduce the amount of expression-dependent variability.
Replication	Each experiment was independently replicated at least 2-3 times (2-3 biological replicates). Replicates were performed independently using similar reagents. All replicates reproduced similar data to those reported.
Randomization	Cells in fields of view from imaging experiments were randomly chosen for quantification. Other forms of randomization were not relevant to our study.
Blinding	No blinding was performed during data collection or analysis, as the differences between measurements of samples was very significant and substantially larger than the error.

Reporting for specific materials, systems and methods

We require information from authors about some types of materials, experimental systems and methods used in many studies. Here, indicate whether each material, system or method listed is relevant to your study. If you are not sure if a list item applies to your research, read the appropriate section before selecting a response.

Materials & experimental systems

n/a	Involved in the study	
☒	☐	Antibodies
☐	☒	Eukaryotic cell lines
☒	☐	Palaeontology and archaeology
☒	☐	Animals and other organisms
☒	☐	Human research participants
☒	☐	Clinical data
☒	☐	Dual use research of concern

Methods

n/a	Involved in the study	
☒	☐	ChIP-seq
☒	☐	Flow cytometry
☒	☐	MRI-based neuroimaging

Eukaryotic cell lines

Policy information about cell lines

Cell line source(s)	HEK293T (ATCC), COS-7 (ATCC)
Authentication	None of the cell lines used were authenticated
Mycoplasma contamination	Not recently tested for mycoplasma contamination
Commonly misidentified lines (See ICLAC register)	No commonly misidentified lines were used.
